# Differential distribution of steroid hormone signaling networks in the human choroid-retinal pigment epithelial complex

**DOI:** 10.1186/s12886-022-02585-7

**Published:** 2022-10-20

**Authors:** Sydney M. Galindez, Andrew Keightley, Peter Koulen

**Affiliations:** 1 School of Medicine, Vision Research Center, Department of Ophthalmology, University of Missouri – Kansas City School of Medicine, 2411 Holmes St, Kansas City, MO 64108 USA; 2 Department of Biomedical Sciences, University of Missouri – Kansas City School of Medicine, Kansas City, MO USA

**Keywords:** Retina, Choroid, RPE, Retinal pigment epithelium, Steroid receptor, Androgen, Estrogen, Glucocorticoids, Mineralocorticoid

## Abstract

**Background:**

The retinal pigment epithelium (RPE), a layer of pigmented cells that lies between the neurosensory retina and the underlying choroid, plays a critical role in maintaining the functional integrity of photoreceptor cells and in mediating communication between the neurosensory retina and choroid. Prior studies have demonstrated neurotrophic effects of select steroids that mitigate the development and progression of retinal degenerative diseases via an array of distinct mechanisms of action.

**Methods:**

Here, we identified major steroid hormone signaling pathways and their key functional protein constituents controlling steroid hormone signaling, which are potentially involved in the mitigation or propagation of retinal degenerative processes, from human proteome datasets with respect to their relative abundances in the retinal periphery, macula, and fovea.

**Results:**

Androgen, glucocorticoid, and progesterone signaling networks were identified and displayed differential distribution patterns within these three anatomically distinct regions of the choroid-retinal pigment epithelial complex. Classical and non-classical estrogen and mineralocorticoid receptors were not identified.

**Conclusion:**

Identified differential distribution patterns suggest both selective susceptibility to chronic neurodegenerative disease processes, as well as potential substrates for drug target discovery and novel drug development focused on steroid signaling pathways in the choroid-RPE.

## Background

The retinal pigment epithelium (RPE) is a layer of pigmented cells that lies between the neurosensory retina and the underlying choroid comprised of the fenestrated choriocapillaris. The location of the RPE allows it to interact with both the neurosensory retina and extra-ocular systems of the body. Given this location, the RPE plays a critical role in mediating the functional communication between the neurosensory retina and the choroid and is also critical to maintaining the functional integrity of photoreceptors [1].

The RPE secretes protective cytokines and growth factors onto the neurosensory retina and conveys waste products to the blood via the choriocapillaris, protecting photoreceptors from ambient insult and also contributes to establishing the immune privilege of the eye by creating a blood-retinal barrier through its tight junctions [1, 2]. The photoreceptors endure daily photo-oxidative damage, which deteriorates the lipid, protein, and opsin contents of the disks. The RPE mediates the essential process of receptor turnover through the phagocytosis of aged photoreceptor disks residing in the photoreceptor outer segments in a diurnal fashion. Nascent phagosomes mature and are resolved within a few hours of the entry of light by healthy RPE, with nutrients and metabolic waste recycled back to photoreceptors or eliminated through the fenestrated choriocapillaris to maintain homeostasis of the retina [3].

The proximally associated choriocapillaris is also critical to maintaining the functional integrity of photoreceptors. These specialized capillaries supply the highly metabolically active photoreceptors and are responsible for removing waste products of metabolism [4].

Given the critical roles these structures play in the visual system, loss of functional integrity of either of these structures could lead to the development of retinal disease.

Understanding the proteomic landscape of the choroid-RPE could provide insight into the pathogenesis of retinal disease and also shed light onto the differential regional susceptibility of the choroid-RPE to the development of disease [5].

Steroids exert neurotrophic effects and may mitigate the development and progression of retinal degenerative disease via an array of mechanisms. Previous studies have shown that ocular tissues express steroid receptors and have demonstrated the neuroprotective effects of several steroids including those of estrogen and glucocorticoids [6]. Here we survey the choroid-RPE proteome dataset for neurosteroids and their pathway constituents from the Skeie and Mahajan, 2014 study, highlighting select pathways potentially involved in neuroprotection and discuss the clinical implications of their relative abundance in regions of the choroid-RPE [5].

## Methods

An initial literature review of androgen, estrogen, mineralocorticoid, glucocorticoid, and progesterone signaling in the central nervous system was conducted in order to develop a list of steroid hormone receptors and proteins involved in steroid signaling in the central nervous system (CNS) that could then be surveyed for from prior proteomic analysis datasets of the choroid-RPE complex. This initial protein list consisted primarily of steroid receptors of the classical and non-classical signaling pathways. Identification of mediators in androgen, estrogen, mineralocorticoid, glucocorticoid, or progesterone signaling in the choroid-RPE complex in proteomic analysis datasets then guided a second and more focused literature review. The aim of the second and more focused literature review was to develop a more comprehensive list of proteins involved in the steroid signaling pathways previously identified in the first screen and to identify other related signaling pathways for which to survey for in the selected proteome dataset.

The proteome dataset from which steroid proteins would be identified during the initial screen of steroid protein receptors was from the Skeie and Mahajan, 2014 study [5]. Skeie and Mahajan characterized the proteomic landscape of the human choroid-RPE complex, identifying proteins expressed in the fovea, macula, and periphery. The study identified over four thousand unique proteins in the choroid-RPE complex from the eyes of one man and two women with no known history of ocular disease and whom were all at least in their eighth decade of life. The ocular tissue was obtained within five hours of death. Skeie and Mahajan exploited a multidimensional chromatographic method to obtain deep proteomic coverage, strong anion exchange fractionation followed by online acidic reversed-phase liquid chromatography (LC)-tandem mass spectrometry (MS). Protein identifications were made using X!!Hunter (against the reference library available in 2010 in the global proteome machine), and X!!tandem. For quantitation, the relative abundance of each identified protein in the fovea, macula, and periphery was based on MS/MS spectral counting in the fovea, macula, and periphery datasets, normalized on the basis on total spectral counts (hits) per sample.

Once a classical or non-classical receptor of a steroid was identified in the Skeie and Mahajan [5], dataset, a literature review was conducted to create a more comprehensive list of protein constituents involved in the respective steroid’s classical and non-classical signaling pathways. The final protein list for each steroid hormone is not intended to be an all-encompassing list of proteins involved in all previously identified steroid-mediated signaling pathways, but rather is a list highlighting select pathways and pathway mediators. A guiding set of criteria was used when choosing steroid signaling pathways to highlight in this study. While some identified pathways may not meet all the criteria, they were highlighted in order to either stimulate further discussion and/or because future research may suggest their relevance to steroid signaling in ocular tissue. The following guiding criteria consisted of: (1) the signaling pathway has been demonstrated to be mediated by steroid proteins or are signaling pathways that mediate the effects of steroid hormones, (2) the signaling pathway was identified in the retina or other central nervous system tissue, and (3) priority was given to steroid signaling pathways that have been demonstrated to be involved in either neuroprotective or degenerative processes.

Once the second literature review was completed and a list of steroid signaling pathways and their constituents was completed, these identified proteins were then cross-referenced with the tandem mass spectrometry (MS/MS) data provided by the Skeie and Mahajan [5], study. For the purposes of discussion, some pathway members may be included since it is possible that absent proteins may exist at an abundance below detection threshold in the current datasets.

Proteins that are involved in steroid hormone signaling and that are indeed localized to the choroid-RPE complex are not discussed here if they were either not identified in the proteome dataset or if they were not identified from this study’s literature review.

Here, we highlight proteins that have been previously identified as mediators of steroid hormone signaling that were also identified in the Skeie and Mahajan [5], choroid-RPE complex tissue proteome analysis. We further highlight the relative abundance of these proteins in the choroid-RPE complex and discuss their potential implications in the pathogenesis and treatment of various retinopathies.

## Results

After conducting a literature review of androgen, estrogen, mineralocorticoid, progesterone, and glucocorticoid signaling, an initial list of proteins to survey for in the proteomic dataset of the Skeie and Mahajan [5], study was developed. This list consisted primarily of classical and non-classical receptors of steroids: nuclear receptor subfamily 3 group C member 2 (NR3C2), estrogen receptor 1 (ESR1), estrogen receptor 2 (ESR2), estrogen related receptor alpha (ESRRA), estrogen related receptor beta (ESRRB), estrogen related receptor gamma (ESRRG), G protein-coupled estrogen receptor 1 (GPER1), progesterone receptor (PGR), progesterone receptor membrane component 1 (PGRMC1), progesterone receptor membrane component 2 (PGRMC2), progestin and adipoQ receptor family member 7 (PAQR7), progestin and adipoQ receptor family member 8 (PAQR8), progestin and adipoQ receptor family member 5 (PAQR5), progestin and adipoQ receptor family member 6 (PAQR6), progestin and adipoQ receptor family member 9 (PAQR9), nuclear receptor subfamily 3 group C member 1 (NR3C1), androgen receptor (AR), G protein-coupled receptor class C group 6 member A (GPRC6A), solute carrier family 39 member 9 (SLC39A9), cytochrome B5 domain containing 2 (CYB5D2), gamma-aminobutyric acid type A receptor subunit alpha1 (GABRA1), gamma-aminobutyric acid type A receptor subunit alpha2 (GABRA2), gamma-aminobutyric acid type A receptor subunit alpha3 (GABRA3), gamma-aminobutyric acid type A receptor subunit alpha4 (GABRA4), gamma-aminobutyric acid type A receptor subunit alpha5 (GABRA5), gamma-aminobutyric acid type A receptor subunit alpha6 (GABRA6), and neudesin neurotrophic factor (NENF) (refer to Table 1 for further protein information and references).

**Table 1:** Summary of proteins surveyed for and identified in the Skeie and Mahajan [5], choroid-RPE proteome analysis. The relative abundance of proteins in regions of the choroid-RPE complex is represented by the average peptide hits in the periphery, macula, and fovea. Note, this MS/MS data is provided by the Skeie and Mahajan [5], study (Key: P: Involved in progesterone signaling; A: Involved in androgen signaling; G: Involved in glucocorticoid signaling; E: Involved in estrogen signaling; M: Involved in mineralocorticoid signaling; Y: Yes, identified in dataset; N: No, not identified in dataset). Gene names and abbreviations were verified with the GeneCards database [7]

	Steroid(s)	Pathway(s)	Further Pathway/Protein Information	Gene name	Protein Name	Identified in the Skeie and Mahajan [5], dataset (Y/N)	Average peptide hits in periphery	Average peptide hits in macula	Average peptide hits in fovea
1	P, A	Non-classical Progesterone Pathway	PI3K-AKT pathway	AKT2	AKT Serine/Threonine Kinase 2	**Y**	13.66666667	0	1.333333333
2	A	Classical & Non-classical Androgen Pathway	–	AR	Androgen Receptor	**Y**	0	0	0.666666667
3	G	Non-classical Glucocorticoid Pathway	–	EGFR	Epidermal Growth Factor Receptor	**Y**	9.333333333	12.66666667	19.33333333
4	G	Classical Glucocorticoid Pathway	–	FLT1	Fms Related Receptor Tyrosine Kinase 1	**Y**	31.33333333	2	2.333333333
5	P	Non-classical Progesterone Pathway	–	FYN	FYN Proto-Oncogene, Src Family Tyrosine Kinase	**Y**	9.666666667	12	9.666666667
6	P, A, G	Non-classical Progesterone/Androgen/Glucocorticoid Pathway	GABA Subunit	GABRA1	Gamma-Aminobutyric Acid Type A Receptor Subunit Alpha1	**Y**	8	0	0
7	P, A, G	Non-classical Progesterone/Androgen/Glucocorticoid Pathway	GABA Subunit	GABRA2	Gamma-Aminobutyric Acid Type A Receptor Subunit Alpha2	**Y**	0	8.666666667	6.666666667
8	P, A, G	Non-classical Progesterone/Androgen/Glucocorticoid Pathway		GABRB2	Gamma-Aminobutyric Acid Type A Receptor Subunit Beta2	**Y**	0	9.333333333	8.333333333
9	G	Non-classical Glucocorticoid Pathway	–	GRB2	Growth Factor Receptor Bound Protein 2	**Y**	15	12.33333333	16
10	P	Non-classical Progesterone Pathway	–	GSK3B	Glycogen Synthase Kinase 3 Beta	**Y**	1.333333333	3.333333333	6.333333333
11	G	Non-classical Glucocorticoid Pathway	–	HRAS	HRas Proto-Oncogene, GTPase	**Y**	3	13.33333333	8.666666667
12	G	Classical Glucocorticoid Pathway	–	KDR	Kinase Insert Domain Receptor	**Y**	4	3	0
13	G	Non-classical Glucocorticoid Pathway	–	MAP2K1	Mitogen-Activated Protein Kinase Kinase 1	**Y**	1	6.333333333	3.666666667
14	P, A, G	Non-classical Progesterone/Androgen/Glucocorticoid Pathway	ERK1/2 pathway	MAPK1	Mitogen-Activated Protein Kinase 1	**Y**	32	20.33333333	28.33333333
15	P, A, G	Non-classical Progesterone/Androgen/Glucocorticoid Pathway	ERK1/2 pathway	MAPK3	Mitogen-Activated Protein Kinase 3	**Y**	29.33333333	19.33333333	24.66666667
16	P	Non-classical Progesterone Pathway	–	NENF	Neudesin Neurotrophic Factor	**Y**	0	0	4.666666667
17	G	Classical Glucocorticoid Pathway	GILZ signaling	NFATC2	Nuclear Factor Of Activated T Cells 2	**Y**	0	0.666666667	1.666666667
18	G	Classical Glucocorticoid Pathway	GILZ signaling	NFKB1	Nuclear Factor Kappa B Subunit 1	**Y**	0	0.666666667	0
19	G	Classical Glucocorticoid Pathway	GILZ signaling	NFKB2	Nuclear Factor Kappa B Subunit 2	**Y**	0	2	0
20	G	Classical Glucocorticoid Pathway	–	NR3C1	Nuclear Receptor Subfamily 3 Group C Member 1	**Y**	0	0	0.666666667
21	P	Non-classical Progesterone Pathway	Mediator of several pathways	PGRMC1	Progesterone Receptor Membrane Component 1	**Y**	36	29.66666667	48.66666667
22	P	Non-classical Progesterone Pathway	Binding partner for PGRMC1	PGRMC2	Progesterone Receptor Membrane Component 2	**Y**	0	15.33333333	17.66666667
23	P	Non-classical Progesterone Pathway	PI3K-AKT pathway	PIK3CB	Phosphatidylinositol-4,5-Bisphosphate 3-Kinase Catalytic Subunit Beta	**Y**	59.66666667	0	0
24	P	Non-classical Progesterone Pathway	PI3K-AKT pathway	PIK3R1	Phosphoinositide-3-Kinase Regulatory Subunit 1	**Y**	1	0	1.333333333
25	P	Non-classical Progesterone Pathway	–	PRKCA	Protein Kinase C Alpha	**Y**	126.6666667	5	7.666666667
26	P	Non-classical Progesterone Pathway	–	PRKCB	Protein Kinase C Beta	**Y**	97.66666667	4.333333333	6.333333333
27	P	Non-classical Progesterone Pathway	–	PRKCD	Protein Kinase C Delta	**Y**	1	3.333333333	2.333333333
28	P	Non-classical Progesterone Pathway	–	PRKCI	Protein Kinase C Iota	**Y**	0.666666667	3.666666667	0
29	G	Non-classical Glucocorticoid Pathway	–	RAF1	Raf-1 Proto-Oncogene, Serine/Threonine Kinase	**Y**	0	1.666666667	0
30	P, G	Non-classical Progesterone/Glucocorticoid Pathway	–	RELA	RELA Proto-Oncogene, NF-KB Subunit	**Y**	0	0.666666667	0
31	A	Non-classical Androgen Pathway	–	RPS6KA1	Ribosomal Protein S6 Kinase A1	**Y**	4.666666667	6	3
32	A	Non-classical Androgen Pathway	–	RPS6KA2	Ribosomal Protein S6 Kinase A2	**Y**	2	2.333333333	1
33	A	Non-classical Androgen Pathway	–	RPS6KA3	Ribosomal Protein S6 Kinase A3	**Y**	4.333333333	3.333333333	4.666666667
34	P	Non-classical Progesterone Pathway	Binding partner for PGRMC1	SERBP1	SERPINE1 MRNA Binding Protein 1	**Y**	7.666666667	11.33333333	13.33333333
35	A	Non-classical Androgen Pathway	–	SHBG	Sex Hormone Binding Globulin	**Y**	18	14.33333333	9.666666667
36	G	Classical Glucocorticoid Pathway	GILZ signaling	SMAD2	SMAD Family Member 2	**Y**	6.666666667	6.666666667	12.33333333
37	G	Classical Glucocorticoid Pathway	GILZ signaling	SMAD3	SMAD Family Member 3	**Y**	7.666666667	1	12.66666667
38	G	Non-classical Glucocorticoid Pathway	–	SRC	SRC Proto-Oncogene, Non-Receptor Tyrosine Kinase	**Y**	14	9.333333333	11.66666667
39	G	Classical Glucocorticoid Pathway	GILZ signaling	TGFB1	Transforming Growth Factor Beta 1	**Y**	1.333333333	1.666666667	1.666666667
41	P, A	Non-classical Progesterone Pathway	PI3K-AKT pathway	AKT1	AKT Serine/Threonine Kinase 1	**N**	**–**	**–**	**–**
42	P, A	Non-classical Progesterone Pathway	PI3K-AKT pathway	AKT3	AKT Serine/Threonine Kinase 3	**N**	**–**	**–**	**–**
43	A	Non-classical Androgen Pathway	–	BAD	BCL2 Associated Agonist Of Cell Death	**N**	**–**	**–**	**–**
44	P	Classical & Non-classical Progesterone Pathways	Neurotrophic protein	BDNF	Brain Derived Neurotrophic Factor	**N**	**–**	**–**	**–**
45	G	Classical Glucocorticoid Pathway	GILZ signaling	CCL2	C-C Motif Chemokine Ligand 2	**N**	**–**	**–**	**–**
46	G	Classical Glucocorticoid Pathway	GILZ signaling	CCL3	C-C Motif Chemokine Ligand 3	**N**	**–**	**–**	**–**
47	P	Non-classical Progesterone Pathway	–	CX3CR1	C-X3-C Motif Chemokine Receptor 1	**N**	**–**	**–**	**–**
48	P	Non-classical Progesterone Pathway	–	CREBBP	CREB Binding Protein	**N**	**–**	**–**	**–**
49	P	Non-classical Progesterone Pathway	–	CYB5D2	Cytochrome B5 Domain Containing 2	**N**	**–**	**–**	**–**
50	P	Non-classical Progesterone Pathway	–	EP300	E1A Binding Protein P300	**N**	**–**	**–**	**–**
51	E	Estrogen Signaling	–	ESR1	Estrogen Receptor 1	**N**	**–**	**–**	**–**
52	E	Estrogen Signaling	–	ESR2	Estrogen Receptor 2	**N**	**–**	**–**	**–**
53	E	Estrogen Signaling	–	ESRRA	Estrogen Related Receptor Alpha	**N**	**–**	**–**	**–**
54	E	Estrogen Signaling	–	ESRRB	Estrogen Related Receptor Beta	**N**	**–**	**–**	**–**
55	E	Estrogen Signaling	–	ESRRG	Estrogen Related Receptor Gamma	**N**	**–**	**–**	**–**
56	P	Non-classical Progesterone Pathway	–	FGF2	Fibroblast Growth Factor 2	**N**	**–**	**–**	**–**
57	G	Classical Glucocorticoid Pathway	GILZ signaling	FOXP3	Forkhead Box P3	**N**	**–**	**–**	**–**
58	P,A,G	Estrogen Signaling	–	GPER1	G Protein-Coupled Estrogen Receptor 1	**N**	**–**	**–**	**–**
59	P,A,G	Non-classical Androgen Pathway	–	GPRC6A	G Protein-Coupled Receptor Class C Group 6 Member A	**N**	**–**	**–**	**–**
60	P,A,G	Non-classical Progesterone/Androgen/Glucocorticoid Pathway	GABA Subunit	GABRA3	Gamma-Aminobutyric Acid Type A Receptor Subunit Alpha3	**N**	**–**	**–**	**–**
61	P,A,G	Non-classical Progesterone/Androgen/Glucocorticoid Pathway	GABA Subunit	GABRA4	Gamma-Aminobutyric Acid Type A Receptor Subunit Alpha4	**N**	**–**	**–**	**–**
62	P,A,G	Non-classical Progesterone/Androgen/Glucocorticoid Pathway	GABA Subunit	GABRA5	Gamma-Aminobutyric Acid Type A Receptor Subunit Alpha5	**N**	**–**	**–**	**–**
63	P,A,G	Non-classical Progesterone/Androgen/Glucocorticoid Pathway	GABA Subunit	GABRA6	Gamma-Aminobutyric Acid Type A Receptor Subunit Alpha6	**N**	**–**	**–**	**–**
64	P,A,G	Non-classical Progesterone/Androgen/Glucocorticoid Pathway	GABA Subunit	GABRB1	Gamma-Aminobutyric Acid Type A Receptor Subunit Beta1	**N**	**–**	**–**	**–**
65	P,A,G	Non-classical Progesterone/Androgen/Glucocorticoid Pathway	GABA Subunit	GABRB3	Gamma-Aminobutyric Acid Type A Receptor Subunit Beta3	**N**	**–**	**–**	**–**
66	P,A,G	Non-classical Progesterone/Androgen/Glucocorticoid Pathway	GABA Subunit	GABRG1	Gamma-Aminobutyric Acid Type A Receptor Subunit Gamma1	**N**	**–**	**–**	**–**
67	P,A,G	Non-classical Progesterone/Androgen/Glucocorticoid Pathway	GABA Subunit	GABRG2	Gamma-Aminobutyric Acid Type A Receptor Subunit Gamma2	**N**	**–**	**–**	**–**
68	P,A,G	Non-classical Progesterone/Androgen/Glucocorticoid Pathway	GABA Subunit	GABRG3	Gamma-Aminobutyric Acid Type A Receptor Subunit Gamma3	**N**	**–**	**–**	**–**
69	P,A,G	Non-classical Progesterone/Androgen/Glucocorticoid Pathway	GABA Subunit	GABRR1	Gamma-Aminobutyric Acid Type A Receptor Subunit Rho1	**N**	**–**	**–**	**–**
70	E	Non-classical Androgen Pathway	–	GABRR2	Gamma-Aminobutyric Acid Type A Receptor Subunit Rho2	**N**	**–**	**–**	**–**
71	A	Non-classical Androgen Pathway	–	GABRR3	Gamma-Aminobutyric Acid Type A Receptor Subunit Rho3	**N**	**–**	**–**	**–**
72	G	Classical Glucocorticoid Pathway	GILZ signaling	IL1R1	Interleukin 1 Receptor Type 1	**N**	**–**	**–**	**–**
73	G	Classical Glucocorticoid Pathway	GILZ signaling	IL17RC	Interleukin 17 Receptor C	**N**	**–**	**–**	**–**
74	G	Classical Glucocorticoid Pathway	GILZ signaling	JUN	Jun Proto-Oncogene, AP-1 Transcription Factor Subunit	**N**	**–**	**–**	**–**
75	P	Non-classical Progesterone Pathway	–	KEAP1	Kelch Like ECH Associated Protein 1	**N**	**–**	**–**	**–**
76	P	Non-classical Progesterone Pathway	–	MAFF	MAF BZIP Transcription Factor F	**N**	**–**	**–**	**–**
77	P	Non-classical Progesterone Pathway	–	MAFG	MAF BZIP Transcription Factor G	**N**	**–**	**–**	**–**
78	P	Non-classical Progesterone Pathway	–	MAFK	MAF BZIP Transcription Factor K	**N**	**–**	**–**	**–**
79	P	Non-classical Progesterone Pathway	Involved in PGRMC1-mediated BDNF release	MAPK7	Mitogen-Activated Protein Kinase 7	**N**	**–**	**–**	**–**
80	G	Classical Glucocorticoid Pathway	GILZ signaling	NFATC1	Nuclear Factor Of Activated T Cells 1	**N**	**–**	**–**	**–**
81	G	Classical Glucocorticoid Pathway	GILZ signaling	NFATC3	Nuclear Factor Of Activated T Cells 3	**N**	**–**	**–**	**–**
82	G	Classical Glucocorticoid Pathway	GILZ signaling	NFATC4	Nuclear Factor Of Activated T Cells 4	**N**	**–**	**–**	**–**
83	G	Classical Glucocorticoid Pathway	GILZ signaling	NFAT5	Nuclear Factor Of Activated T Cells 5	**N**	**–**	**–**	**–**
84	P	Non-classical Progesterone Pathway	–	NFE2L2	Nuclear Factor, Erythroid 2 Like 2	**N**	**–**	**–**	**–**
85	M	Classical Mineralocorticoid Pathway	–	NR3C2	Nuclear Receptor Subfamily 3 Group C Member 2	**N**	**–**	**–**	**–**
86	P	Non-classical Progesterone Pathway	**–**	PIK3CA	Phosphatidylinositol-4,5-Bisphosphate 3-Kinase Catalytic Subunit Alpha	**N**	**–**	**–**	**–**
87	P	Non-classical Progesterone Pathway	**–**	PIK3CD	Phosphatidylinositol-4,5-Bisphosphate 3-Kinase Catalytic Subunit Delta	**N**	**–**	**–**	**–**
88	P	Non-classical Progesterone Pathway	**–**	PIK3CG	Phosphatidylinositol-4,5-Bisphosphate 3-Kinase Catalytic Subunit Gamma	**N**	**–**	**–**	**–**
89	P	Non-classical Progesterone Pathway	**–**	PGR	Progesterone Receptor	**N**	**–**	**–**	**–**
90	P	Non-classical Progesterone Pathway	**–**	PAQR5	Progestin And AdipoQ Receptor Family Member 5	**N**	**–**	**–**	**–**
91	P	Classical Progesterone Pathway	**–**	PAQR6	Progestin And AdipoQ Receptor Family Member 6	**N**	**–**	**–**	**–**
92	P	Non-classical Progesterone Pathway	PI3K-AKT pathway	PAQR7	Progestin And AdipoQ Receptor Family Member 7	**N**	**–**	**–**	**–**
93	P	Non-classical Progesterone Pathway	PI3K-AKT pathway	PAQR8	Progestin And AdipoQ Receptor Family Member 8	**N**	**–**	**–**	**–**
94	P	Non-classical Progesterone Pathway	–	PAQR9	Progestin And AdipoQ Receptor Family Member 9	**N**	**–**	**–**	**–**
95	G	Classical Glucocorticoid Pathway	GILZ signaling	PRKCZ	Protein Kinase C Zeta	**N**	**–**	**–**	**–**
96	G	Classical Glucocorticoid Pathway	GILZ signaling	REL	REL Proto-Oncogene, NF-KB Subunit	**N**	**–**	**–**	**–**
97	G	Classical Glucocorticoid Pathway	GILZ signaling	RELB	RELB Proto-Oncogene, NF-KB Subunit	**N**	**–**	**–**	**–**
98	G	Classical Glucocorticoid Pathway	–	SMAD4	SMAD Family Member 4	**N**	**–**	**–**	**–**
99	P	Non-classical Progesterone Pathway	–	TSC22D3	TSC22 Domain Family Member 3 (GILZ)	**N**	**–**	**–**	**–**
100	P	Non-classical Progesterone Pathway	PI3K-AKT pathway	VEGFA	Vascular Endothelial Growth Factor A	**N**	**–**	**–**	**–**
101	A	Non-classical Androgen Pathway	Membrane androgen receptor	SLC39A9	Solute Carrier Family 39 Member 9	**N**	**–**	**–**	**–**

Of these proteins, the following were identified in the Skeie and Mahajan [5], dataset: AR, NR3C1, PGRMC1, PGRMC2, GABRA1, GABRA2, and NENF (refer to Table 1 for further protein information and references). These identified proteins are involved in androgen, glucocorticoid, and progesterone signaling. This initial survey then guided the second and more focused literature review of androgen, glucocorticoid, and progesterone signaling in the retina and other CNS tissues to develop a more comprehensive list of pathways involved in steroid signaling and their respective constituents. As previously described, select pathways were identified in this study, using the criteria described in the methods section. The list of proteins that was surveyed for in the Skeie and Mahajan [5], dataset can be found in Table 1 and consists of one-hundred-and-one unique proteins. While this list does not include all proteins involved in all identified pathways, it aims to identify key pathway mediators.

The proteome dataset from the Skeie and Mahajan [5], study was then surveyed to identify proteins from the second protein list. Of the one-hundred-and-one unique proteins surveyed for, 39 were identified in the Skeie and Mahajan [5], proteome dataset. The average number of peptides identified in the periphery, macula, and fovea from the MS/MS data of the Skeie and Mahajan [5], study were noted for each protein.

### Androgen signaling

**Fig. 1:**
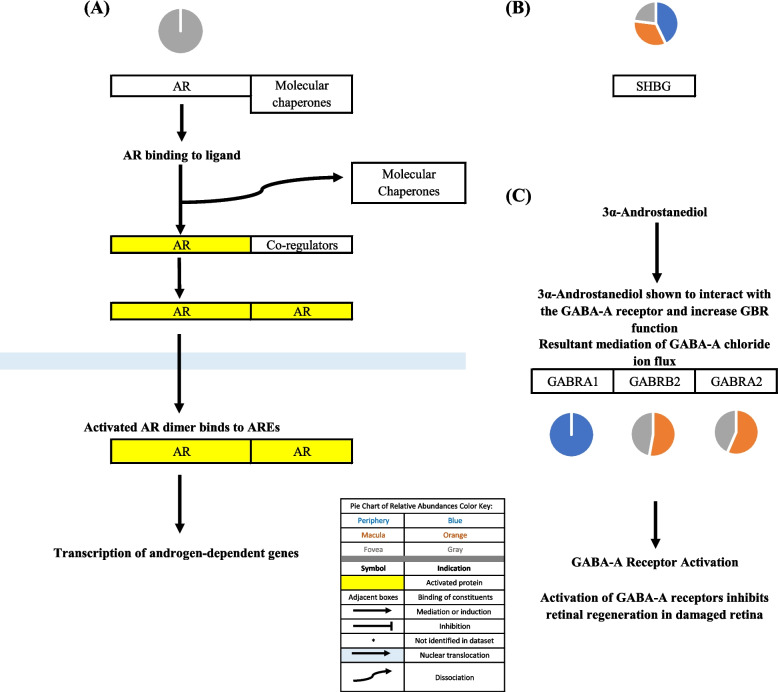
**A** Classical androgen receptor (AR) signaling pathway. When unbound, AR is localized in the cytoplasm associated with molecular chaperones. Binding of AR to its appropriate ligand induces a conformational change that leads to the dissociation of AR from the protein chaperones. AR then interacts with co-regulators that facilitate nuclear targeting and dimerization. AR typically forms a homodimer and is then translocated across the nuclear membrane where it binds to androgen response elements (AREs) and induces the transcription of  androgen-dependent genes [9, 10]. **B** Sex hormone binding globulin (SHBG) binds androgens and estrogens and transports these steroids in the systemic circulation. SHBG is believed to also be involved in steroid hormone signaling [11]. **C** 3α-Androstanediol has been shown to interact with the γ-aminobutyric acid (GABA)-A receptor and increase GABA receptor (GBR) function, mediating the GABA-A chloride ion flux demonstrated in Long Evans rats [8]. GABA-A receptor activation has been shown to inhibit retinal regeneration in damaged retina in zebrafish [12]. Pie charts adjacent to proteins represent the protein’s relative abundance in the periphery (blue), macula (orange), and fovea (gray) based on the mass spectrometry data provided by the Skeie and Mahajan [5] study

**Fig. 2:**
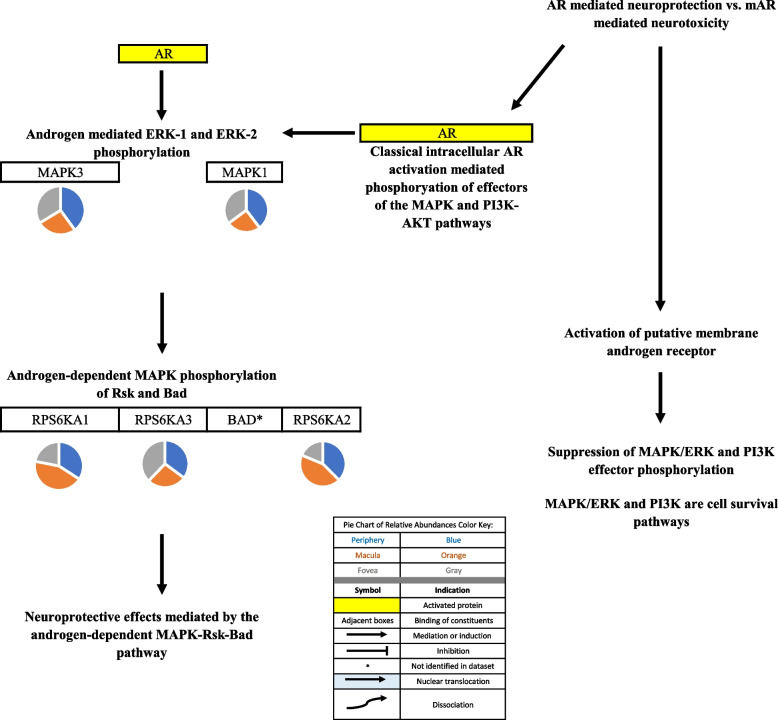
Evidence suggest that androgens can mediate either neuroprotective or neurotoxic effects. Classical intracellular AR activation mediated phosphorylation of effectors of the MAPK and PI3K-AKT pathways while activation of putative membrane androgen receptors mediated suppression of phosphorylation of MAPK and PI3K-AKT pathway effectors in C6 cells and cortical astrocytes [13, 14]. These studies suggest that consideration of androgen receptor type predominance in select tissues may be warranted when developing androgen pathway targeted interventions. Pie charts adjacent to proteins represent the protein’s relative abundance in the periphery (blue), macula (orange), and fovea (gray) based on the mass spectrometry data provided by the Skeie and Mahajan [5] study

Androgen signaling exerts both neuroprotective and neurotoxic effects in CNS tissue via classical and non-classical signaling pathways (as described in the “Androgen signaling” section of the discussion). The classical androgen receptor, AR, was identified in the fovea but not in the macula or periphery. Sex hormone binding globulin was identified in the periphery, macula, and fovea. Androgen metabolites interact with the γ-Aminobutyric acid type A (GABAA) receptor [8]. GABAA receptor subunits were identified in the periphery, macula, and fovea. Solute Carrier Family 39 Member 9 (SLC39A9) and G Protein-Coupled Receptor Class C Group 6 Member A (GPRC6A), non-classical androgen receptors, were not identified in the proteome dataset. Further information regarding protein constituents of androgen signaling can be found in Table 1. The above-mentioned signaling pathways are depicted in Figs. 1 and 2.

### Glucocorticoid signaling

**Fig. 3:**
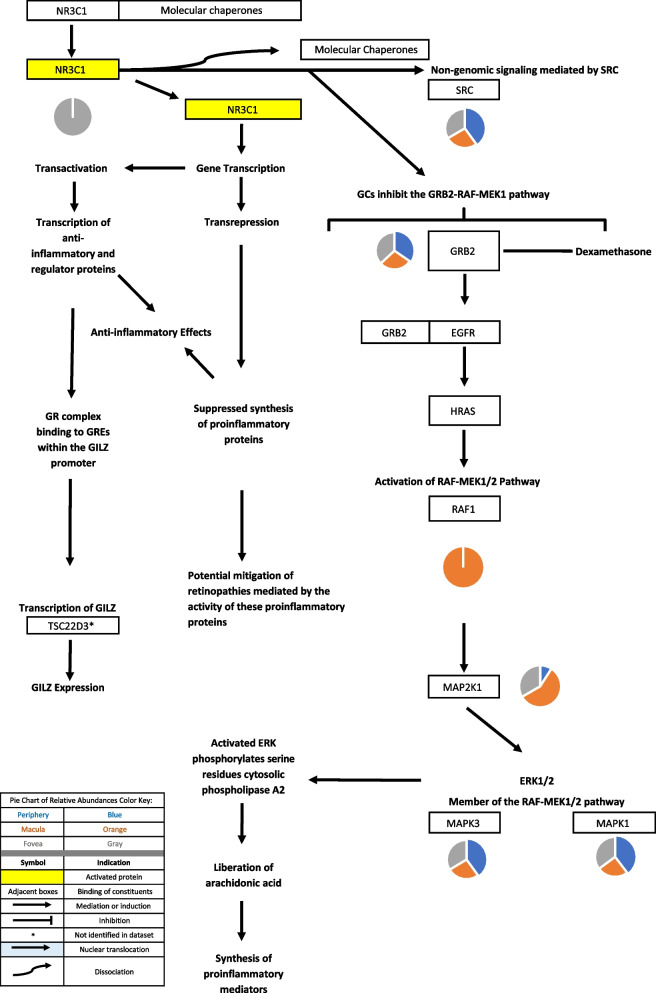
When the classical glucocorticoid receptor (GR) is unbound to its ligand, it is bound to a multiprotein complex of molecular chaperones. Upon binding to its appropriate ligand, GR undergoes conformational changes, dissociating from the molecular chaperones. As a result, its nuclear localization signal is exposed and the GR is translocated to the nucleus [17, 18]. The genomic actions of glucocorticoids include either transcriptional activation of genes or the transcriptional repression of genes. Transactivation consists of activation of glucocorticoid response elements (GREs) and transcription of anti-inflammatory and regulator proteins [19, 20]. The GR complex can also bind to GREs within the GILZ promoter, initiating the transcription of the Glucocorticoid-induced leucine zipper protein (GILZ), a mediator of glucocorticoid effects [21]. Transrepression consists of the GR complex binding to transcription factor subunits, preventing their association to DNA and their co-activators [19, 20];. Glucocorticoids (GCs) are also involved in non-genomic signaling. GCs have been shown to inhibit the GRB2-RAF-MEK1 pathway in A549 cells by blocking GRB2 recruitment [22]. The pathway leads to the downstream phosphorylation of cytosolic phospholipase A2 and consequent liberation of arachidonic acid and pro-inflammatory proteins [22, 23]. Pie charts adjacent to proteins represent the protein’s relative abundance in the periphery (blue), macula (orange), and fovea (gray) based on the mass spectrometry data provided by the Skeie and Mahajan [5] study

**Fig. 4:**
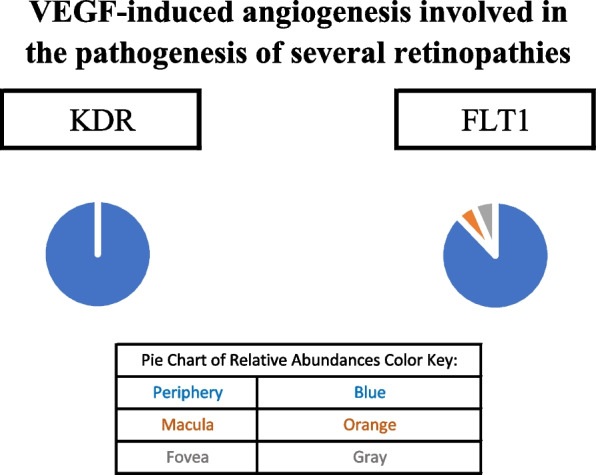
VEGF has been demonstrated to be produced and secreted by RPE cells. VEGF-induced angiogenesis is involved in the pathogenesis of several retinopathies including AMD, retinopathy of prematurity, and neovascular glaucoma [24]. VEGFR-1 and VEGFR-2 both bind to VEGF-A [25]. VEGFR-2 exhibits broad pro-angiogenic effects while VEGFR-1 has a greater affinity to its ligand but is more tightly regulated with less potent activity [26]. Pie charts adjacent to proteins represent the protein’s relative abundance in the periphery (blue), macula (orange), and fovea (gray) based on the mass spectrometry data provided by the Skeie and Mahajan [5] study

**Fig. 5:**
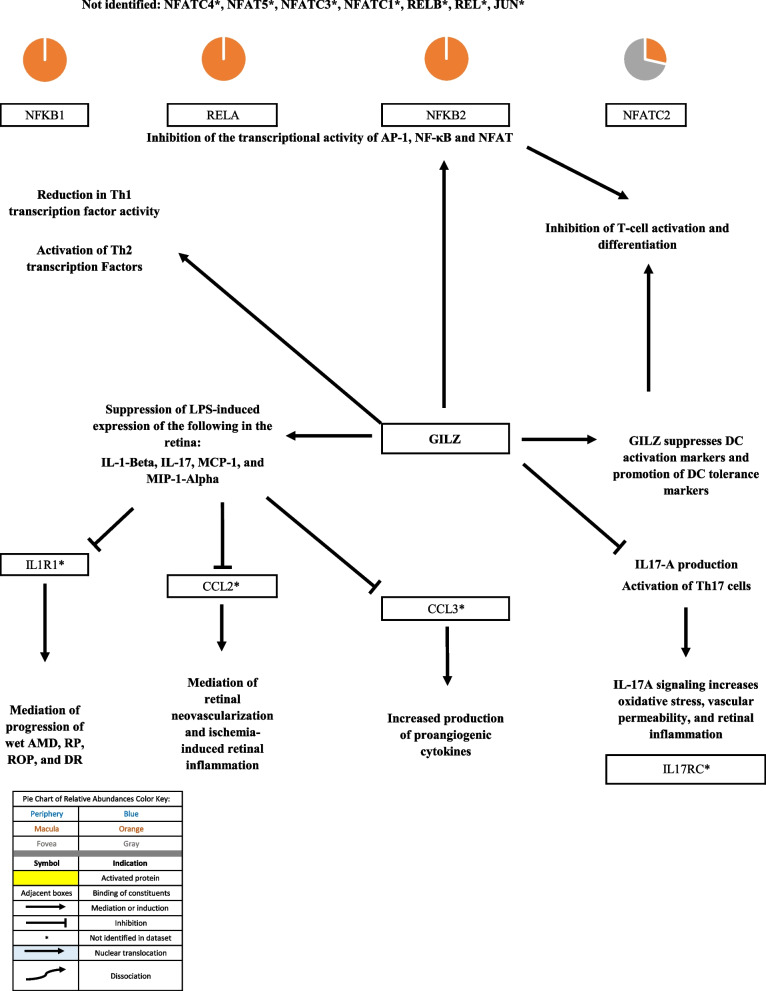
The GILZ protein is an immune-modulating protein that exerts numerous effects. GILZ inactivates several, transcription factors that regulate T-cell activation and differentiation, including NF-kB [21, 27]. GILZ was also shown to reduce Th1 transcription factor activity and activates Th2 transcription factors in T-cells from GILZ transgenic mice [28]. GILZ was shown to suppress lipopolysaccharide (LPS)-induced expression of several interleukins and chemokines in the retina in male Sprague-Dawley rats with endotoxin-induced uveitis [29]. Suppression of these cytokines inhibited macrophage Inflammatory protein-1 alpha (MIP-1a) which is expressed in the hypoxic inner retina in a C57BL/6 model for ischemia-induced retina neovascularization and may attract microglia that induce angiogenesis, suppressed monocyte chemoattractant protein-1 activity which has also been shown to mediate retinal neovascularization, and as well as supressed interleukin-beta [30-34]. GILZ suppresses dendritic cell (DC) activation markers and promoted tolerance markers, inhibiting T-cell activation and differentiation [21, 35, 36]. Finally, GILZ has been shown to inhibit interleukin 17 alpha production and subsequent activation of Th17 cells in a murine model for streptozotocin-induced diabetes which increases retinal inflammation [37]. Pie charts adjacent to proteins represent the protein’s relative abundance in the periphery (blue), macula (orange), and fovea (gray) based on the mass spectrometry data provided by the Skeie and Mahajan [5] study

**Fig. 6:**
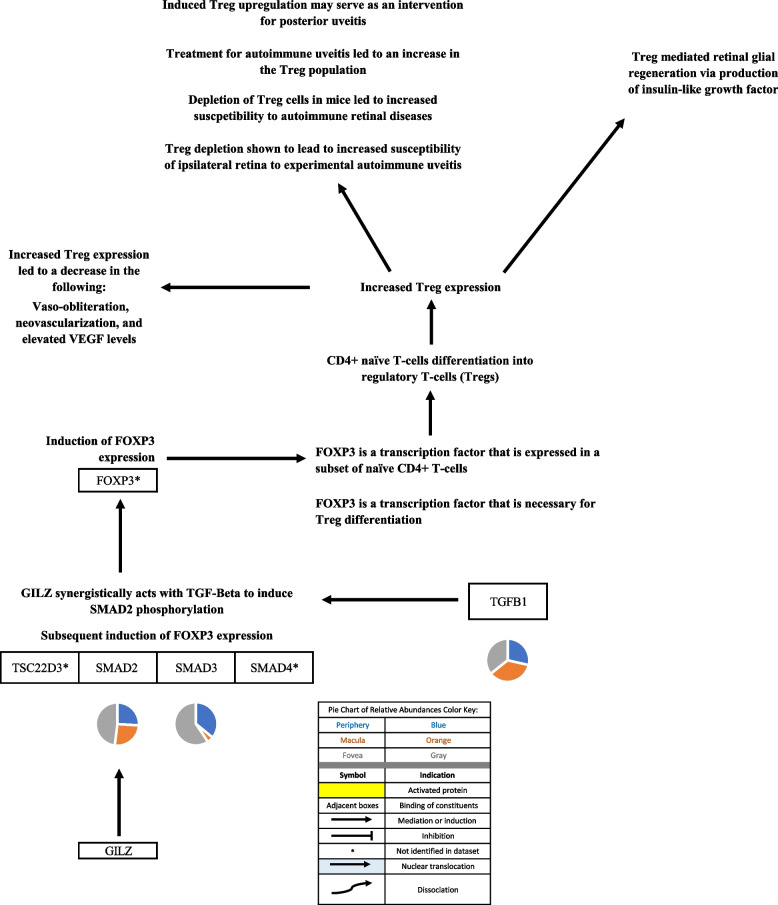
GILZ synergistically acts with TGF-beta to induce SMAD2 phosphorylation and subsequently induce FOXP3 expression [38]. FOXP3 is a transcription factor expressed in a subset of naïve CD4+ T-cells that is necessary for Treg differentiation [39, 40]. Regulatory T-cells (Treg) upregulation may serve as an intervention for posterior uveitis as a study demonstrated that Treg depletion leads to increased susceptibility of the ipsilateral retina to experimental autoimmune uveitis [41, 42]. Tregs were also shown to mediate retinal glial regeneration via production of insulin-like growth factor [43]. Pie charts adjacent to proteins represent the protein’s relative abundance in the periphery (blue), macula (orange), and fovea (gray) based on the mass spectrometry data provided by the Skeie and Mahajan [5] study

**Fig. 7:**
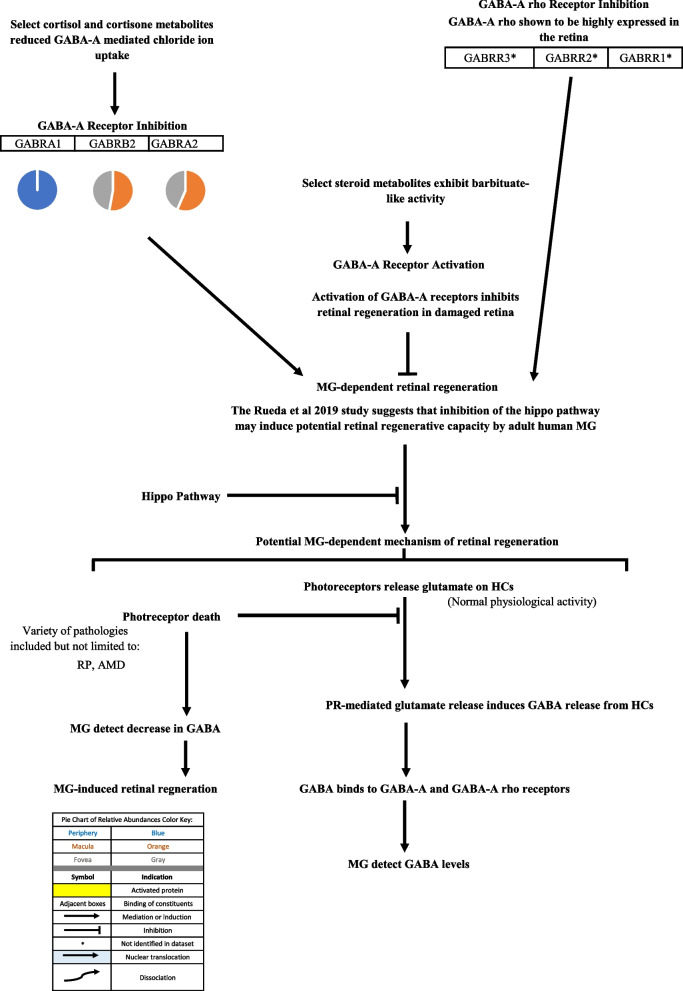
Retinal regeneration model. Inhibition of GABA-A receptors in the zebrafish retina induced muller glial (MG)-dependent regeneration [12, 44]. The Kent et al., 2021 [45] study results further support decreasing GABA levels as a potential mechanism for retinal regeneration via MG de-differentiation and generation of progenitor cells. This regenerative pathway, however, may be inhibited by the hippo pathway in mammals. Inhibition of the hippo pathway may induce potential retinal regenerative capacity by adult human MG [46]. Select steroid metabolites have been demonstrated to inhibit GABA-A activity while others exhibited barbiturate-like activity [15, 16]. GABA-A rho has also been shown to be highly expressed in the retina and was thus surveyed for in this study [47]. Pie charts adjacent to proteins represent the protein’s relative abundance in the periphery (blue), macula (orange), and fovea (gray) based on the mass spectrometry data provided by the Skeie and Mahajan [5] study

Glucocorticoids modulate inflammation via both genomic and non-genomic signaling (as described in the “Glucocorticoid signaling” section of the discussion). The classical glucocorticoid (GC) receptor, NR3C1, was identified in the fovea but not in the periphery or macula. Glucocorticoid-induced leucine zipper protein (GILZ), a protein product of the classical GC transcription pathway, was not identified in the proteome dataset. GC metabolites regulate GABAA receptor activity [15, 16]. As previously mentioned, GABAA receptor subunits were identified in the periphery, macula, and fovea. Further information regarding protein constituents of glucocorticoid signaling can be found in Table 1. The above-mentioned signaling pathways are depicted in Figs. 3, 4, 5, 6 and 7.

### Progesterone signaling

**Fig. 8:**
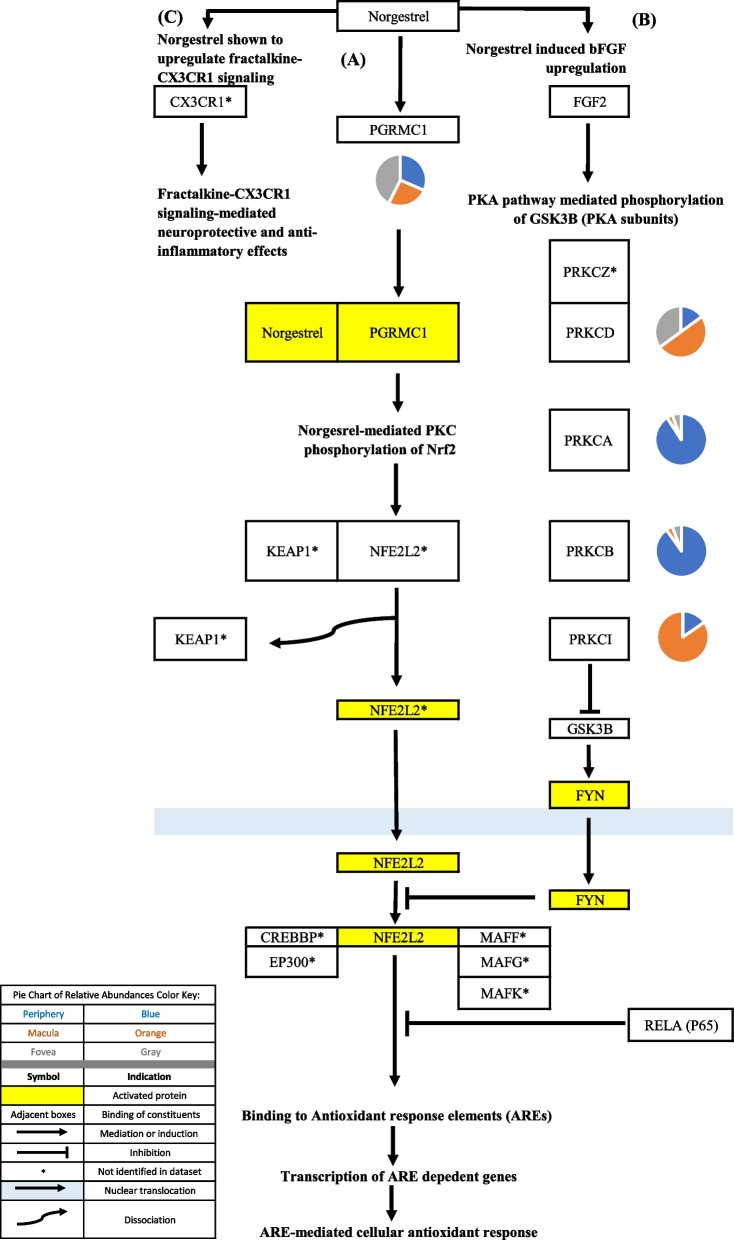
**A** Non-classical progestin signaling. PGRMC1 has been found to be highly expressed in the 661 W photoreceptor cell line, RPE, and MG [52, 53]. Norgestrel has been shown to exhibit antioxidant properties, preventing photoreceptor damage in a model for RP via increasing the expression of Nrf2, which in response to oxidative stress, binds to DNA anti-oxidant response elements and initiates the transcription of cytoprotective genes [54-56]. Huang et al. [57] suggested that it is the phosphorylation of Nrf2 by protein kinase C that induces Nrf2’s nuclear translocation and anti-oxidant effects. **B** Norgestrel exhibits neuroprotective properties in stressed photoreceptor-like cells and retinal explants via upregulating basic fibroblast growth factor (bFGF) activity via a protein kinase A pathway-dependent mechanism. bFGF phosphorylates and inactivates glycogen synthase kinase 3-beta (GSK3B), preventing the dysregulation of the Nrf2 defense system via preventing the phosphorylating FYN which induces the nuclear export and degradation of Nrf2 [56, 58, 59]. **C** Norgestrel upregulates fractalkine-CX3CR1 signaling in 661 W cells and C57 explants and fractalkine signaling which mediates norgestrel cytoprotection via reduction of inflammatory cytokine production in rd10 microglia [60]. Pie charts adjacent to proteins represent the protein’s relative abundance in the periphery (blue), macula (orange), and fovea (gray) based on the mass spectrometry data provided by the Skeie and Mahajan [5] study

**Fig. 9:**
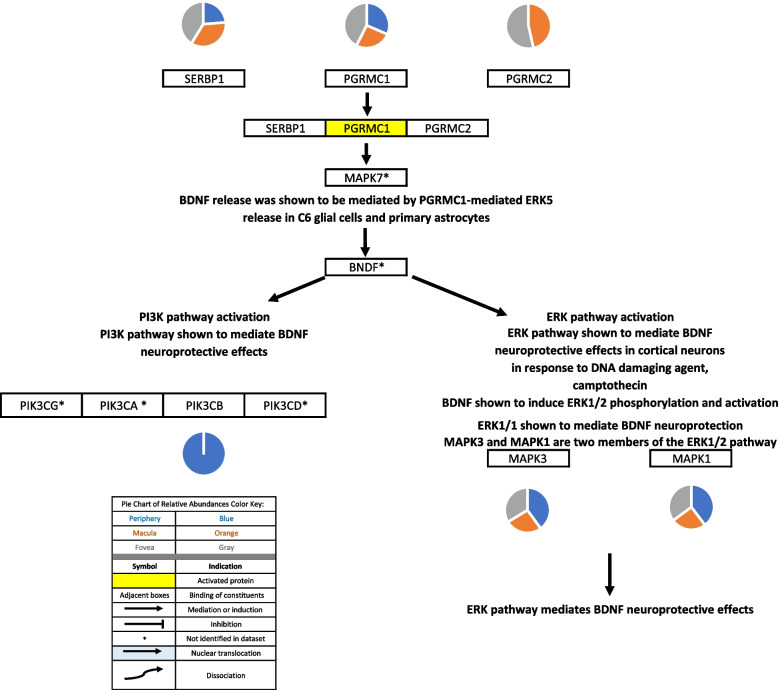
PGRMC1 signaling. SERBP1 and PGRMC2 may serve as binding partners for PGRMC1 [48, 49]. Progesterone induces BDNF expression via PGRMC1-dependent ERK5 signaling which mediates progesterone’s cytoprotective effects in C6 glial cells and primary astrocytes [61]. The PI3K and ERK pathways have been shown to mediate BDNF neuroprotective effects in response to serum deprivation and DNA damaging agents such as camptothecin in cortical neurons [62]. Pie charts adjacent to proteins represent the protein’s relative abundance in the periphery (blue), macula (orange), and fovea (gray) based on the mass spectrometry data provided by the Skeie and Mahajan [5] study

Progesterone-mediated signaling occurs via both classical and non-classical pathways in CNS tissue (as described in the “Progesterone signaling” section of the discussion). Of all the identified classical and non-classical steroid receptors, PGRMC1 was most highly expressed in the periphery, macula, and fovea. PGRMC1 binding partners, PGRMC2 and SERPINE1 MRNA Binding Protein 1 (SERBP1), were also identified in the proteome dataset [48, 49]. The classical progesterone receptor, PGR, was not identified in the proteome dataset. Progesterone membrane receptors that are members of the progestin and adipoQ receptor family were also not identified in the proteome dataset. Progesterone interacts with the GABAA receptor [50]. GABAA receptor subunits were identified in the periphery, macula, and fovea. Cytochrome B5 domain containing 2 was not identified in the proteome dataset. NENF suggested to be a non-classical progesterone receptor, was only identified in the fovea [51]. Further information regarding protein constituents of progesterone signaling can be found in Table 1. The above-mentioned signaling pathways are depicted in Figs. 8 and 9.

### Estrogen signaling

Estrogens exert variable retinal neuroprotective effects via nuclear estrogen receptor signaling, membrane receptor signaling, as well as through interactions with other intracellular cytoprotective signaling pathways (as described in the in the “Estrogen signaling” section of the discussion). The estrogen receptors, estrogen receptor 1, estrogen receptor 2, estrogen related receptor alpha, estrogen related receptor beta, estrogen related receptor gamma, and G protein-coupled estrogen receptor 1 were not identified in the proteome dataset.

### Mineralocorticoid signaling

The mineralocorticoid receptor has been identified previously in the retina and can exert its effects after aldosterone-mediated activation (as described in the “Mineralocorticoid signaling” section of the discussion). The classical mineralocorticoid receptor, NR3C2, was not identified in the proteome dataset.

## Discussion

The study identified mediators of the progesterone, androgen, and glucocorticoid signaling pathways, all which may have implications in the pathogenesis or treatment of retinopathies. Classical and non-classical estrogen or mineralocorticoid receptors were not identified in the Skeie and Mahajan [5], proteome dataset, while androgen, glucocorticoid, and progesterone receptors and associated proteins were identified.

Although the present study did not identify estrogen or mineralocorticoid receptors, this lack of identification does not indicate the absence of these receptors in the choroid-RPE complex as the analyzed proteome datasets focused only on identified protein mediators of steroid signaling pathways. Previous studies, for example, have reported the presence of estrogen receptors in the choroid-RPE complex, including estrogen receptor (ER)-alpha and ER-beta [63]. Estrogen’s neuroprotective effects in the RPE and in other CNS tissues has also been well studied [63-66].

### Androgen signaling

The classical androgen receptor, AR, was identified in the fovea, but not in the macula or periphery. AR has been previously identified in the choroid-RPE complex [67]. Androgens are important modulators of neuronal viability, demonstrating both neuroprotective and neurotoxic effects [68-71].

Androgen-induced neurotoxic effects have been shown to be mediated by AR. Cunningham and colleagues demonstrated testosterone induced apoptosis of N27 neurons, a dopaminergic cell line, via activation of the protein kinase C-delta pathway and that this was dependent upon AR activation [70]. Chronic exposure to androgens has also been suggested to promote mitochondrial dysfunction. Testosterone increases mitochondrial dysfunction in N27 neurons, increasing the levels of reactive oxygen species (ROS) and reducing levels of thiol in N27 cells. These effects were suggested to be dependent upon AR activation [70]. Mitochondrial functioning is important for the regulation of cell death. Mitochondria both generate ROS, which is important for activation of the apoptosis pathway, and generates antioxidant enzymes to protect cells from apoptosis [72]. Appropriate mitochondrial function is necessary to sustain RPE function. Choroid-RPE complex mitochondria are especially vulnerable to oxidative damage and is believed to be intimately involved in the pathogenesis of retinal degeneration [73]. While androgen induced mitochondrial damage has been demonstrated in N27 cells, further research is needed to determine if this activity would be present in the retina. If such an interaction were to be identified in the RPE, this may have implications for the pathogenesis of retinopathies mediated by mitochondrial damage that affect the fovea. Evidence suggests that mitochondrial dysfunction in the choroid-RPE is intimately associated with the development and progression of age-related macular degeneration (AMD) [73].

AR has also played an important role in mediating androgens’ neuroprotective effects [69, 71]. Hammond and colleagues demonstrated that serum deprivation-mediated neuronal apoptosis of human primary neurons was either eliminated entirely or significantly reduced at physiological concentrations of testosterone enanthate. This neuroprotective effect did not require aromatization into estrogens and the neuroprotective effects were prevented when treated with flutamide, suggesting that androgen neuroprotection is mediated via AR [74].

AR has also been involved in mediating non-genomic signaling via activation of second messengers. Activation of AR activates phospholipase C (PLC) via activation of an inhibitory G-protein (GPi). PLC activation may then lead to the rise in intracellular inositol 1,4,5-triphosphate, release of calcium from the sarcoplasmic reticulum, and subsequent regulation of the RAS/MEK/ERK pathway (RAS = “rat sarcoma virus,” a family of GTPases that regulate cell growth and differentiation; MEK = MAPK/ERK kinase; ERK = extracellular-signal regulated kinase) [71]. If this specific interaction between AR and GPi were to be identified in the RPE, it may have important implications for choroid-RPE integrity in the setting of AR dysfunction. Calcium has been demonstrated to regulate the RAS/MEK/ERK pathway, which in many studied tissues, mediates cell survival by regulating the activity of the pro-apoptotic molecules Bad and Bcl-2 (BCL2 = BCL2 apoptosis regulator; bad = BCL2 associated agonist of cell death) [75-77]. ERK pathway activation appears to inhibit apoptosis in most tissues, although it has been reported that calcium mediated ERK pathway activation is involved in stress-activated apoptosis of lens epithelial cells [77]. Nguyen and colleagues also demonstrated that androgen-induced neuroprotection in hippocampal neurons was mediated via the androgen-dependent ERK-Rsk (ribosomal s6 kinase)-bad pathway [78].

Gatson and colleagues showed that in addition to phosphorylation of ERK, an effector of the ERK neuroprotective pathway, AR activation also mediates the phosphorylation of AKT, an effector of the phosphoinositide-3-kinase–protein kinase B/Akt (PI3K-PKB/Akt) signaling pathway. The PI3K-Akt signaling pathway is important in regulating cell growth and viability [13].

Androgens interact with non-classical androgen receptors [71]. Expression of sex hormone binding globulin (SHBG), which binds circulating androgens and estrogens was identified in the fovea, macula, and periphery in the proteome dataset. SHBG not only regulates androgen and estrogen bioavailability but also acts as a modulator of unique signaling pathways.

SHBG, for example, inhibits estrogen’s anti-apoptotic effects in MCF-7 cells, a breast cancer cell line. Estrogen phosphorylation of extracellular signal-regulated kinase 1/2 (ERK1/2) mediates estrogen induced anti-apoptotic effects. SHBG, when bound to the SHBG membrane receptor (SHBG-R) and subsequently to its steroid ligand inhibits estrogen’s anti-apoptotic effects through the elevation of cyclic adenosine monophosphate (cAMP) levels. Elevated cAMP, which has been previously demonstrated to suppress ERK1/2 activity, was suggested to reduce the ERK-dependent anti-apoptotic effects of estrogen [79, 80]. Both androgens and estrogens are involved in the formation of the SHBG-receptor(R)—SHBG—steroid complex and subsequent induction of cAMP synthesis and resultant downstream signaling. Estrogens and androgens, however, participate in this complex formation in a cell-type specific manner [81].

The SHBG receptor has also been identified in endometrial cell membranes, placental tissue, and prostatic membranes. To our knowledge, the SHBG-R gene has not yet been characterized [81]. It is possible that the SHBG-R may be present in the RPE but further research is needed to elucidate this. If the SHBG-R is identified in the choroid-RPE, its anti-estrogenic actions could inhibit estrogen-mediated neuroprotective effects in the retina. Again, further research would be needed to demonstrate this interaction in the retina.

Androgen metabolites interact with the γ-Aminobutyric acid type A (GABAA) receptor. GABAA receptor subunits were identified in the proteome dataset. γ-aminobutyric acid type A receptor subunit alpha1 (GABRA1) was identified only in the periphery, while γ-aminobutyric acid type A receptor subunit alpha2 (GABRA2) and γ-aminobutyric acid type A receptor subunit beta2 (GABRB2) were identified in the in the macula and fovea but not in the periphery. Identification of these subunits is important in determining the structure of the GABAA receptor(s) in the RPE and subsequent studying of its specific activity. GABAA receptors are composed of five subunits around a central ion pore [82]. There are fifteen different protein subunits that can form different isoforms of the GABAA receptor. Homomeric isoforms as well as isoforms with different subunits are possible. By identifying GABAA receptors subunits, one may be able to deduce the possible structure of GABAA receptor(s) in regions of the choroid-RPE.

The androgen metabolite, 3α-Androstanediol, interacts with the GABAA receptor and increases the activity of the GABA-receptor complexes (GBRs), resulting in increased chloride ion flux [8]. This may have significance in the development of future therapeutics aimed at treating retinal degeneration as demonstrated by a study that explored the mechanism of GABA-regulated retinal regeneration in zebrafish [12]. GABAA activation was demonstrated to inhibit retinal regeneration in damaged retinas of zebrafish while inhibition of GABAA stimulated regeneration in undamaged retinas via induction of Müller glia (MG) proliferation. MG are able to detect decreased levels of GABA and respond by undergoing differentiation, serving as progenitors in retinal regeneration. Humans have MG in the retina, however, MG-mediated retinal regeneration is inhibited in mammals [12]. Rueda and colleagues demonstrated that the Hippo pathway, by repressing the activity of a transcriptional co-factor called YAP (yes-associated protein), blocked the mammalian MG-mediated retinal regeneration process and that inhibition of the Hippo pathway could stimulate the potentially latent regenerative activity of mammalian MG [46].

Gatson and colleagues provided insight for the differential actions of androgens, those being either mediating neuroprotection or neurotoxicity. It was suggested that the differential actions of androgens in various tissues may be due to the predominance of either AR or membrane androgen receptors [13]. AR activation leads to the activation of both ERK and PI3K-AKT neuroprotective pathways while activation of a putative androgen membrane receptor suppressed ERK and PI3K-AKT pathway activation in primary cortical astrocytes [14]. While the findings of Gatson and colleagues accurately describe the androgen activity in certain neural tissues, it is important to note that AR activation also has injurious effects on cells, as demonstrated by Cunningham and colleagues [70].

Further research elucidating the predominance of certain androgen receptors, as well as research that further explores androgen signaling in the retina may be helpful in the development of androgen-targeted therapeutics aimed at treating retinal degeneration.

### Glucocorticoid signaling

Glucocorticoids are widely used as therapeutics for their anti-inflammatory effects [83]. Excessive inflammation is involved in the development and progression of a wide array of diseases including ocular diseases such as AMD, retinitis pigmentosa (RP), and glaucoma [30, 84, 85].

The glucocorticoid classical receptor, NR3C1, was detected in the fovea but not in the macula or periphery. While the lack of detection of NR3C1 in the macula and periphery does not necessitate its absence in these regions, it may suggest that even if NR3C1 is present in these regions, it may be more abundant in the fovea.

Glucocorticoids (GCs) exert their effects via the classical or genomic pathway, as well as via non-genomic (non-classical) pathways. In the classical pathway GCs either transcriptionally activate genes (transactivation) or transcriptionally repress genes (transrepression) [17, 86]. The transactivation pathway activates anti-inflammatory and regulatory proteins [19, 20, 86]. Glucocorticoid-induced leucine zipper (GILZ) is an especially important protein that mediates GC anti-inflammatory effects [21]. GILZ was not identified in the proteome dataset but this may be due to the fact that the samples of ocular tissue were taken from individuals with no known ocular disease and therefore, glucocorticoid receptor activation and resultant increase in GILZ levels would not have been observed.

GILZ, a major effector of the GC transactivation pathway, mediates anti-inflammatory effects via numerous mechanisms. GILZ suppresses the activity of pro-inflammatory transcription factors including NFkB-p65 (subunit of NF-kappa-B transcription complex), activator protein-1 (AP-1), NF-κB (nuclear factor-kappa B), and NFATC2 [21, 27]. These transcription factors were identified in the proteome dataset in macula and fovea, but not in the periphery.

GILZ-mediated glucocorticoid effects suppress lipopolysaccharide (LPS)-induced retinal inflammation, inhibit T-cell differentiation, and increase T-regulatory (Treg) cell expression [21, 29, 35, 38-40]. Further, GILZ may suppress T-cell interleukin (IL)-17A production [87]. This is may be clinically important as IL-17A signaling exacerbates vascular permeability, retinal inflammation, and oxidative stress. This was demonstrated in a murine model of streptozotocin-diabetes. It is worth noting that while the IL-17A receptor (IL17RC) was not identified in the proteome dataset, it is constitutively expressed in neural and vascular cells of the retina and has been identified in photoreceptors [37].

A potentially valuable aspect of GILZ is that while it mediates GC effects, its activity may not produce the unfavorable GC-associated metabolic effects [88]. A better understanding of GILZ activity would be useful in the potential future development of therapeutics that mitigate inflammation in ocular tissue while avoiding the GC-induced metabolic effects.

While GILZ activation, as well as activation of other anti-inflammatory and regulatory proteins is hallmark of the transactivation route of GC classical signaling, many GC effects are also mediated by the transrepression pathway. In transrepression, transcription factor activity is inhibited, suppressing the synthesis of pro-inflammatory cytokines including IL-1, IL2, IL-6, IL-8, vascular endothelial growth factor (VEGF), cyclooxygenase-2 (COX-2), prostaglandins, tumor necrosis factor (TNF), and interferon (IFN)-gamma. Transrepression mediated GC anti-inflammatory effects are more clinically desirable and produce less unfavorable metabolic effects [19, 20]. The transrepression mediated activity of GC has important implications in a variety of retinopathies as inflammation is involved in the pathogenesis of AMD, diabetic retinopathy (DR), retinal vein occlusion (RVO), diabetic macular edema, and RP [89-91]. Transrepression-dependent GC activity may also have therapeutic effects in retinopathies that exhibit excessive angiogenesis such as AMD, DR, retinopathy of prematurity, sickle cell retinopathy, neovascular glaucoma, and inherited retinopathies [24]. GC can suppress the transcription of VEGF, which has been demonstrated to be secreted and produced by RPE cells, astrocytes, MG, vascular endothelium, and ganglion cells [19, 24]. In the proteome dataset, receptors to VEGF-A, kinase insert domain receptor (KDR), and Fms related receptor tyrosine kinase 1 (FLT1) were primarily identified in the periphery. FLT1, however, was also identified in the macula and fovea. The localization of these VEGF receptors in the retina may suggest that different regions of the choroid-RPE complex may be more susceptible to pathologic angiogenesis.

NR3C1 may also mediate anti-inflammatory effects via non-genomic mechanisms. GR inhibits the GRB2-RAF-MEK1 pathway (GRB2 = growth factor receptor bound protein 2; RAF = RAF Proto-Oncogene Serine/Threonine-Protein Kinase; MEK1 = Mitogen-Activated Protein Kinase Kinase 1) in A549 cells, a human lung adenocarcinoma cell line, by blocking GRB2 recruitment, preventing its association to phosphorylated EGFR and subsequent downstream signaling [22]. Blockage of this signaling inhibits the liberation of arachidonic acid and subsequent synthesis of pro-inflammatory mediators [92].

Different glucocorticoid metabolites interact with the GABAA receptor and either inhibit or stimulate GABA-mediated chloride ion uptake. The glucocorticoid metabolites, allotetrahydrocortisol, tetrahydrocortisol, allotetrahydrocortisone, tetrahydrocortisone, were demonstrated to inhibit GABAA activity in cortical micro sacs of adult male Wistar rats [15]. 3alpha,5alpha-tetrahydrodeoxycorticosterone exhibits barbiturate like activity, potentiating GABAA-mediated increased chloride ion uptake. This was demonstrated in hippocampal and spinal cord neurons in a murine model [16]. As previously discussed, the GABAA subunits that were identified in the proteome dataset include the GABRA1, GABRA2, and GABRB2 subunits. GABRA1 was only identified in the periphery while GABRA2 and GABRB2 were identified in the macula and fovea. These identifications may provide insight to the structure of the GABAA receptor(s) localized in the choroid-RPE complex. Also as previously mentioned, modulation of GABAA activity may have significance in the future development of therapeutics that stimulate MG-dependent retinal regeneration.

### Progesterone signaling

The classical progesterone receptor, PGR, was not identified in the proteome dataset but has been previously identified in the retina of rd10 mice (rd10, retinal degeneration 10), a model for retinitis pigmentosa, and C57 wild type mice [58]. Upon binding to its steroid ligand, PGR dimerizes, is translocated into the nucleus, and then modulates gene transcription [93]. PGR upregulates the transcription of the neurotrophic protein, brain-derived neurotrophic factor (BDNF) [94]. BDNF serves as a mediator of progesterone induced neuroprotection against a variety of insults and unfavorable ambient conditions including ischemia, trauma, and glutamate toxicity [94-96].

In addition to regulating the transcription of genes through the classical or genomic signaling pathway, progesterone can also interact with non-classical receptors and interact with protein kinases. The non-classical progesterone membrane receptors, PAQR5, PAQR7, PAQR8, were not identified in the dataset, but are expressed in the 661 W photoreceptor cell line [52, 58].

A well-studied progesterone membrane receptor is progesterone receptor membrane component 1 (PGRMC1) and has been previously shown to be expressed in the RPE [53]. Regulation of PGRMC1 activity is crucial for cell viability and has been identified as a regulator of apoptosis. PGRMC1 expression has been demonstrated to be upregulated in the setting of retinal degeneration in the rd10 mouse retina [58]. PGRMC1 has been identified to be co-localized with binding partners progesterone receptor membrane component 2 (PGRMC2) and plasminogen activator inhibitor 1 (SERBP1) [48, 49]. PGRMC1 and SERBP1 were identified in the periphery, macula, and fovea in the proteome dataset. PGRMC2, however, was identified in the macula and fovea but not in the periphery. PGRMC2 does not bind progesterone and its function in progesterone signaling is not as well understood [48, 49].

PGRMC1 was identified in the proteomic dataset and was found to be expressed in the periphery, macula, and fovea. PGRMC1 mediates progesterone induced neuroprotection via numerous mechanisms. One such studied mechanism is PGRMC1-mediated norgestrel induced transcription of antioxidants [54, 97]. In this pathway, PGRMC1, activated by norgestrel (a progestin), induces protein kinase C (PKC) phosphorylation of nuclear factor erythroid 2–related factor 2 (Nrf2) [54]. Phosphorylation of Nrf2 then allows for dissociation of Nrf2 from its cytoplasmic repressor, Kelch-like ECH-associated protein 1 (KEAP1), allowing for Nrf2 to then be translocated into the nucleus, form a heterodimer with a small Maf (sMAF) protein, and then upregulate the transcription of cytoprotective genes [54, 57, 98]. Although Nrf2 and KEAP1 were not identified in the dataset, studies have demonstrated that Nrf2 signaling is present in the RPE. Previous studies have also shown that Nrf2 signaling is impaired in the aging RPE and that compromised Nrf2 antioxidant effects may render the aging RPE to be more vulnerable to oxidative stress-induced damage to the RPE and subsequently develop AMD [99]. Norgestrel also promotes neuroprotection by inhibiting glycogen synthase kinase 3b (GSK3B) signaling [100]. Downstream GSK3B signaling induces the nuclear export and ubiquitination of Nrf2 [59]. Norgestrel inhibits GSK3B signaling via basic fibroblast growth factor and protein kinase A (PKA) [100]. This led to a reduction of inflammatory cytokine production in rd10 microglia [60].

Su and colleagues also demonstrated that PGRMC1 mediated the progesterone-induced release of neurotrophic BDNF in rat C6 glioma cells via activation of the ERK5 signaling pathway [61]. The PI3K-AKT and ERK1/2 pathways may then mediate BDNF neuroprotection as demonstrated in the Harlan Sprague-Dawley rat brain [62, 101]. Members of the PI3K-AKT pathway were identified in the proteome dataset and were found to be expressed in the periphery and macula. Members of the ERK1/2 pathway were identified in the periphery, macula, and fovea.

PGRMC1 also has pro-angiogenic effects. Lange and colleagues demonstrated that PGRMC1 can induce VEGF expression in MG. PGRMC1 was demonstrated to induce calcium influx in MG, leading to PI3K-dependent phosphorylation of protein kinase C and ERK1/2 and downstream transcription of VEGF [102]. While physiologic stimulation of angiogenesis in response to oxygen deprivation may be protective, dysregulation of this VEGF-A expression pathway may lead to pathologic neovascularization in the choroid-RPE.

Progesterone can also interact with another non-classical receptor, the GABAA receptor; Callachan and colleagues demonstrated that select progesterone metabolites can activate the GABAA receptor. This was demonstrated in bovine adrenomedullary chromaffin cells [50]. These findings are consistent with the findings of the Majewska [16] study that showed that progesterone metabolites may be barbiturate-like regulators of GABAA receptor activity.

Visual representations of the identified androgen, glucocorticoid, and progesterone signaling pathways [9, 10, 12-15, 17-47, 52-62, 67-102] are depicted in Figs. 1, 2, 3, 4, 5, 6, 7, 8 and 9.

### Lack of identification of classical and non-classical estrogen and mineralocorticoid receptors

While the present study did not identify estrogen and mineralocorticoid receptors in the dataset, these receptors have been previously identified in ocular tissue [63-66, 103-116].

### Estrogen signaling

Estrogen receptor expression in ocular tissue as well as the local production of estrogens in the retina via androgen aromatization and cholesterol-based synthesis has been described extensively [63-66, 104-114]. ESR1 and ESR2 have been identified in the human RPE-choroid complex and GPER1, a G-protein coupled estrogen receptor, has been localized to the endoplasmic reticulum of CNS cell types including retinal ganglion cells [106, 107, 111-114].

Studies have provided evidence that estrogens may exert neuroprotective effects in the retina, including promoting RPE cell survival during periods of oxidative stress and exerting neuroprotective effects in the setting of glutamate and N-methyl-D-aspartate (NMDA) toxicity [63-66, 104-114]. Estrogens may exert neuroprotective effects via nuclear estrogen receptor signaling, membrane receptor signaling, as well as via its interactions with other intracellular pathways [63]. Jiang and colleagues demonstrated that administration GPER1 agonists mitigated murine Müller cell gliosis and retinal ganglion cell apoptosis in the setting of NMDA neurotoxicity and that these effects may be mediated by the PI3K-AKT signaling pathway [110].

Studies have suggested that estrogen receptor expression levels as well as retinal functioning may be positively associated with higher estrogenic states [113, 115]. This apparent association could possibly explain the lack of identification of estrogen receptors from the proteome dataset. The Skeie and Mahajan [5], study utilized ocular tissue from the eyes of one man and two presumably postmenopausal women who were all at least in their eighth decade of life and may have had estrogen receptor expression levels below the threshold for detection. This, however, is only speculative as further studies would be needed to further explore this apparent association.

### Mineralocorticoid signaling

While the mineralocorticoid nuclear receptor (MR), NR3C2, was not identified in the dataset, it has been previously identified in the retina, including in ganglion cells, RPE cells, and in vascular cells [103].

Studies have provided evidence for the deleterious effects of high aldosterone levels and the overactivation of MR in the retina. Wilkinson-Berka and colleagues identified aldosterone and its associated receptor, NR3C2, in the pathogenesis of retinal vasculopathy in animal models [103]. The study demonstrated that MR antagonism mitigated angiogenesis, reported MR antagonism’s reduction of retinal leukostasis and aldosterone-mediated tubulogenesis, and provided evidence that increased aldosterone levels may compromise the functioning of the antioxidant glutathione system. Studies have also provided evidence that aldosterone-mediated MR overactivation may be implicated in the pathogenesis of retinal edema via upregulation of ion and water channels involved in retinal fluid homeostasis [116].

Further, Wagner and colleagues provided evidence of an intraocular renin-angiotensin system (RAS) that is distinct from the RAS in systemic circulation and found that the renin gene was most highly expressed in the RPE choroidal layer of enucleated human eyes [117]. This system may be an important regulator of intraocular MR activity.

Reasons as to why the nuclear mineralocorticoid receptor was not identified in the Skeie and Mahajan [5], proteome dataset could include protein levels below the detection threshold, selective degradation of the protein, or both.

Future studies that further elucidate the steroid signaling pathways in the choroid-RPE, may provide valuable insight into both regional differences in disease susceptibility and responsiveness to steroid pathway targeted therapies.

## Data Availability

The datasets analyzed during the current study are available in reference [5], Skeie and Mahajan, 2014.

## Abbreviations

ANOVA: Analysis of variance
AMD: Age-Related Macular Degeneration
AR: Androgen receptor
bFGF: Basic fibroblast growth factor
cAMP: Cyclic adenosine monophosphate
CNS: Central nervous system
DR: Diabetic retinopathy
ER: Estrogen receptor
GC: Glucocorticoid
GILZ: Glucocorticoid-induced leucine zipper
GSK3B: Glycogen synthase kinase 3-beta
LC: Liquid chromatography
MG: Müller glia
MS: Mass spectrometry
Nrf2: Nuclear factor erythroid 2-related factor 2
PLC: Phospholipase C
PR: Progesterone receptor
PGRMC1: Progesterone receptor membrane component 1
PGRMC2: Progesterone receptor membrane component 2
ROS: Reactive oxygen species
RP: Retinitis pigmentosa
RPE: Retinal pigment epithelium
RVO: Retinal vein occlusion
SERBP1: Plasminogen activator inhibitor 1
